# Effect of Neutral and Negative Images on Galvanic Skin Response: An Activity for Courses in Forensic, Affective, and Behavioral Neuroscience

**DOI:** 10.59390/001c.154524

**Published:** 2025-12-30

**Authors:** Robert W. Flint, Shania Jagda

**Affiliations:** 1 Life Sciences, Psychology Program Albany College of Pharmacy and Health Sciences; 2 Allied Health Sciences, Public Health Program Albany College of Pharmacy and Health Sciences

**Keywords:** galvanic skin response (GSR), stress, arousal, forensic neuroscience, affective neuroscience, behavioral neuroscience, classroom activity

## Abstract

The galvanic skin response (GSR) has provided important scientific insight in a wide range of contexts and has been used in neuroscience research for many decades. It is important for undergraduate students to understand this versatile technique and its application in areas such as Affective, Behavioral, and Forensic Neuroscience. Participants in this study viewed a slideshow containing negative and neutral images selected from the RADIATE and IAPS databases after being connected to a small portable GSR biofeedback monitor. Images were presented for 7-sec on a computer screen followed by a 20-sec blank screen. Each participant’s highest GSR response during the 7-sec image presentation was recorded. Participants provided a valence rating, using a 5-point Likert scale, immediately after each image was presented. The mean GSR for images rated as negative was significantly higher than the mean GSR for images rated as neutral. Results were discussed with the class prior to the completion of demographic and activity effectiveness questionnaires. All responses were significant on the activity effectiveness questionnaire. Participants reported a better understanding of the use of GSR in neuroscience, considered this activity a valuable experience, and recommended its use in future classes.

The electrodermal response, or galvanic skin response (GSR), has a long history (Widacki, 2015) and is often initially thought of in association with studies on emotion (Horvers et al., 2021; Kreibig et al., 2007), stress (Joshi et al., 2017; Yang et al., 2021), and lying/deception (Lykken, 1959; Orne & Thackray, 1967; Thackray & Orne, 1968). However, its use is far more extensive, and it has become an important variable of study in neuroscience research with both human and non-human primates (see Table 1; Laine et al., 2009). GSR has been used to assess activation of the sympathetic division of the autonomic nervous system in studies on a wide variety of topics ranging from academic performance (Villanueva et al., 2016) to the effects of TV commercials (Vecchiato et al., 2010), and has implicated a role for many different neural structures in primates including the amygdala and sensorimotor, parietal, and cingulate cortex (Gertler et al., 2020; Wood et al., 2014; Zhang et al., 2013)

Many different types of stimuli may produce stress- and/or emotion-induced sympathetic nervous system changes. Human faces have been frequently used to elicit a GSR and faces are particularly relevant stimuli in the fields of Affective and Forensic Neuroscience (Avram et al., 2010; Banks et al., 2012, 2014; Juuse et al., 2024; Stagg et al., 2013). Several databases have been developed to study emotional facial expressions including the Racially Diverse Affective Expression face set (RADIATE, Conley et al., 2018; Tottenham et al., 2009), The City Infant Faces Database (Webb et al., 2018), and the Pictures of Facial Affect database (POFA; Ekman & Friesen, 1976). Similarly, the study of human emotion has resulted in the development of several sets of stimuli for validated assessment. Databases such as the International Affective Picture System (IAPS; Lang et al., 1999), the Nencki Affective Picture System (NAPS; Marchewka et al., 2014), the Geneva Affective Picture Database (GAPED; Dan-Glauser & Scherer, 2011), and the Open Affective Standardized Image Set (OASIS, Kurdi et al., 2017) have been used extensively to study emotion. The development and availability of databases such as these to study facial expressions and emotions also provides an opportunity to develop classroom activities that afford students opportunities to use and experience some of the same stimuli and research protocols that scientists are using to explore these phenomena.

**Table 1.** Representative Studies Using Galvanic Skin Response

Topic	Reference(s)
Aggression	de Looff et al. (2019)
Auditory Threshold	Neri et al. (2022)
Biofeedback Training	Castelletti et al. (2024); Markiewicz & Dobrowolska (2021)
Cognitive Load	Shi et al. (2007)
Drug Use	Ding et al. (2020)
Education	Dong et al. (2025); Villanueva et al. (2016)
Experiencing	Keelin (1973)
Emotion	Chung et al. (2007); Korpal & Jankowiak (2018); Prasolenko et al. (2017)
Epilepsy	Nagai (2011, p. 2019); Sandor et al. (2025)
Guilt	Yu et al. (2017)
Kleptomania	Olbrich et al. (2019)
Hypoglycemic Stress	Patel et al. (2014)
Language	Parker (2007); Pishghadam et al. (2024)
Lying	KreyBig & Krautz (2019)
Maternal Depression	Mareckova et al. (2025)
Meditation	Anand (2014)
Mental Health	Demedts et al. (2023); Gordon et al. (2025); Markiewicz et al. (2022); Radu et al. (2003); Vahey & Becerra (2015)
Movement Toward Reward	Amiez et al. (2003)
Music	Stephenson et al. (2016)
Pain	Painter et al. (1965)
Transcranial Magnetic Stimulation	Cox et al. (2023)
Trauma/PTSD	Machlin et al. (2024); Putica et al. (2023)
TV Commercials	Vecchiato et al. (2010)

Despite the importance of GSR in neuroscience research, there are relatively few published studies addressing its pedagogical use in higher education. Nepal et al. (2018) used GSR as a teaching tool in a physiology lab as an indicator of sympathetic nervous system arousal. In their study they used a subtraction task to manipulate cognitive load, a concept relevant to cognitive neuroscience but of less interest to students in Forensic and/or Affective neuroscience. It is not clear what equipment was used to examine GSR, so the feasibility of adopting the activity for use in undergraduate neuroscience courses is not clear. In a study examining student engagement, McNeal et al. (2020) used Empatica E4 wrist biosensors in a large introductory biology course. They found that the use of biosensors has promising potential as an indicator of student engagement in the classroom, but the activity was not utilized as a means of educating students on the GSR technique.

As interest in neuroscience and its subdisciplines continues to grow, so does the need for classroom activities that help students understand how equipment and techniques are applied in neuroscientific research and applied settings. Utilizing classroom activities may also help students connect with the course material (Flint et al., 2025). GSR is often used as a measurement tool in forensic-related disciplines. For example, Kumar (2012) explored connections between GSR and psychological well-being in criminals convicted of crimes such as murder, rape, and robbery, Loeber et al. (2007) examined skin conductance as a possible predictor of desistance from delinquency, Gatzke-Kopp et al. (2002) examined electrodermal responses in connection with sensation seeking and delinquent behavior, and Brennan et al. (1997) studied skin conductance in males at high risk for criminal behavior.

The objective was to develop an activity that would allow students to better understand the connection between emotional arousal and galvanic skin response. We used stimuli with a negative emotional valence to maximize the likelihood of eliciting a GSR, and because of the high relevance of such stimuli to Affective Neuroscience and the evolving field of Forensic Neuroscience. Relatively inexpensive GSR monitors were used as a means of establishing a reliable activity that would be affordable for most faculty.

## Materials and Methods

### Participants

A total of twenty-one students from one section of Forensic Psychology and one section of Neuropsychology at Albany College of Pharmacy and Health Sciences participated in the GSR activity in the spring of 2025. Of those, 19 were present the day the demographic and activity experience questionnaires were completed. Participant age ranged from 19 to 33 with a mean of 21.3 years. Two participants were in their first year and there were 4 sophomores, 9 juniors, and 4 seniors. Fourteen of the participants indicated their sex/gender status as woman while 4 indicated man, and 1 indicated non-binary. Race and ethnicity revealed that most of the participants (11) were white, 1 indicated Hispanic or Latino, 1 Black or African American, 4 Asian, and 2 indicated two or more races. Participants were offered 10 points of extra credit for participating in the activity. An alternative article critique assignment was made available to any student who did not wish to participate in the GSR activity. Approval of all procedures was obtained from the Institutional Review Board for research with human participants prior to any data collection.

### Apparatus and Materials

A portable Bio-Feedback Monitor-GSR2-Expert from Hypno-Quip Hypnotherapy Equipment UK Ltd. was used to obtain galvanic skin responses (https://www.hypno-quip.co.uk/). This easy-to-use and relatively inexpensive device was equipped with a bio-sensor lead with two terminals. Continuous GSR readings were recorded to the tenth of an ohm.

Research has shown that negative images may produce significant changes in GSR, and the amplitude of the change is reportedly related to the level of arousal induced by the images (Bradley et al., 2001). A total of 16 images were selected (see Table 2 for brief description and stimulus set numbers). Eight images, 4 neutral and 4 negative, were selected from the International Affective Picture System (IAPS; Lang et al., 1999). Eight male face images were also selected from the Racially Diverse Affective Expression (RADIATE) face stimulus set, 4 neutral and 4 negative (Conley et al., 2018; Tottenham et al., 2009). Negative images were selected to elicit stronger GSRs in accordance with the literature. Images were arranged into a PowerPoint slideshow with 1 image per slide and presented using a Dell Latitude 5400 laptop computer connected to an external 24-inch Dell color monitor.

A demographic questionnaire was used to collect descriptive information from the participants, and a short activity questionnaire was created to assess each participant’s perspective regarding the educational value of the GSR activity.

**Table 2.** Arousing and Neutral Stimuli from the RADIATE and IAPS Databases

RADIATE			
Neutral	Stimulus #	Arousing	Stimulus #
Male, Neutral	AM01_NC	Male, Angry	BM03_AO
Male, Neutral	AM07_NC	Male, Angry	AM02_AO
Male, Neutral	BM11_NC	Male, Angry	AM11_AO
Male, Neutral	BM16_NC	Male, Angry	BM02_AO
IAPS			
Neutral	Stimulus #	Arousing	Stimulus #
Electrical Outlet	6150	Dental Surgery	9584
Spoon	7004	Attacking Snake	1052
Book	7090	Bloodied Boxer	8230
Towel	7002	Tarantula on Shoulder	1201

### Procedure

Students from the PI’s Forensic Neuroscience and Neuropsychology classes received a lecture on GSR in relation to the course content, after which they were provided with a brief overview of the research participation opportunity. It was explained that the PI and a research assistant would collect data from individual students, but the hope was to develop an activity in the future where students would have the opportunity to use GSR monitors themselves in class to collect data with their classmates. Students interested in volunteering for the research signed up for a time to come to the PI’s lab to participate. After signing an informed consent form, bio-sensor terminals were connected to the middle phalanges of each participants’ index and middle fingers. Participants were asked to find a comfortable and relaxing position while the procedure was explained. Participants were told that they would watch a PowerPoint slideshow consisting of 16 images, presented full-screen for 7-sec, each followed by a 20-sec white screen with a large black ‘+’ centered on it. Participants were told to focus on the image on the screen of the 24-inch Dell color monitor. The researchers simultaneously watched the slideshow presentation on the laptop and the GSR monitor and recorded the highest GSR value from the monitor during each stimulus presentation. Immediately following each image presentation, after the blank screen appeared, participants were asked to provide a rating of the previous image using a 5-pt Likert scale (1=very negative, 2=negative, 3=neutral, 4=positive, 5=very positive) which the researchers noted on the datasheet. Once the PowerPoint slideshow was completed the bio-sensor terminals were removed from the participant’s fingers and a short debriefing statement was read.

Following GSR data collection, the results were analyzed by the PI and a short PowerPoint presentation was developed. The data analysis procedure and results were then presented to the classes. The general procedure and purpose of the activity was discussed along with the statistical analysis and interpretation of the results. Immediately following the classroom presentation and discussion of the results, participants were asked to complete the demographic and activity effectiveness questionnaires.

## Results

Individual participants’ baseline GSR values at the start of the study were highly variable as were the valence ratings participants provided for each image. To determine whether images with different emotional valences elicited different GSR responses, two mean GSR values were calculated for analysis. The first mean was determined using only the GSR for images each individual participant rated as ‘neutral’ (3 on the Likert scale), thus providing a mean ‘neutral’ GSR. The second mean was calculated using only images each individual participant rated as ‘very negative’ or ‘negative’ (1 or 2 on the Likert scale), providing a ‘negative’ GSR value.

Seventeen of the twenty-one participants had a higher mean GSR for images they had rated as negative in comparison to those they rated as neutral. A paired samples t-test for the mean neutral (M = 57.017, SD = 14.191) and mean negative (M = 57.891, SD = 13.154) GSR values revealed a significantly higher GSR value for negative images [t(20) = 2.34, *p* = .015].

**Figure 1.**
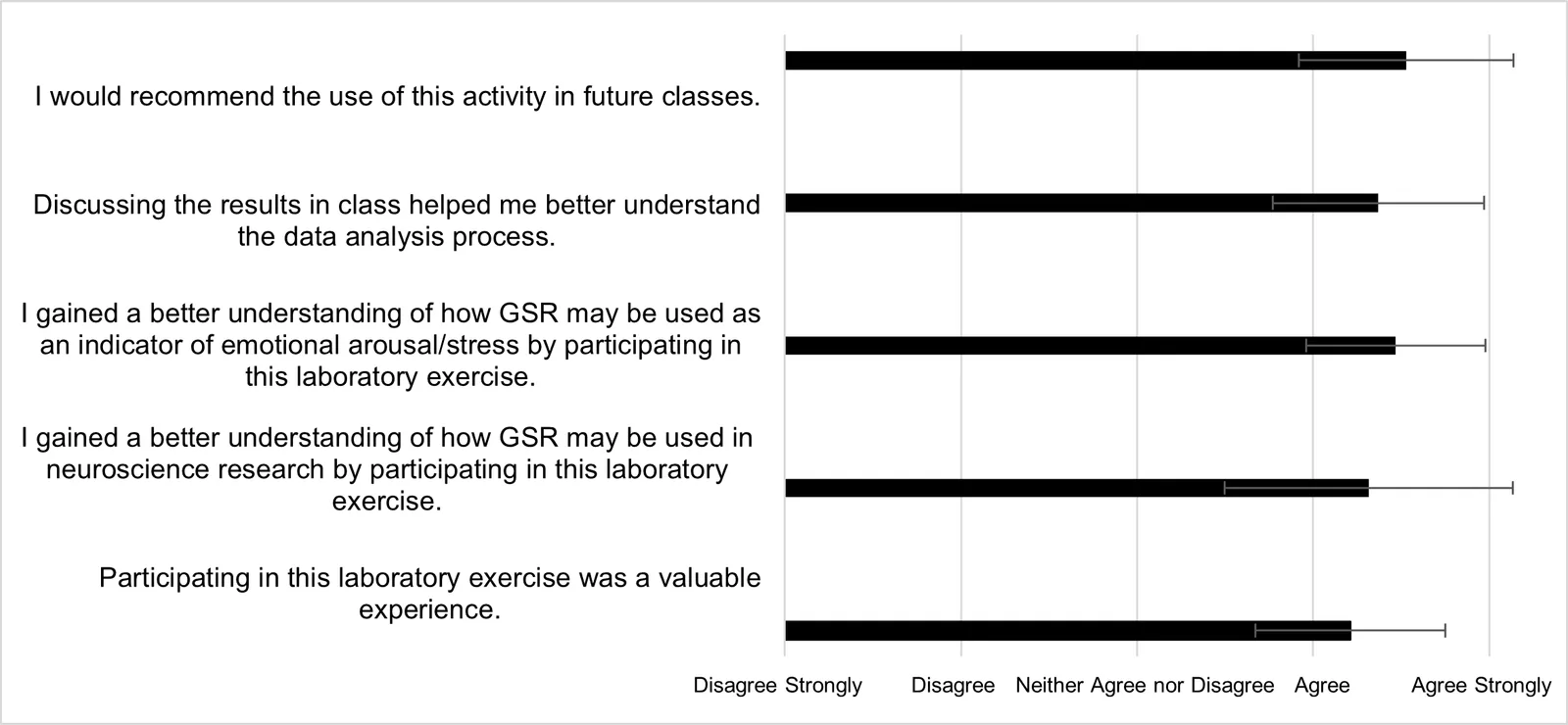
Activity Effectiveness Questionnaire. Mean responses and standard deviations on the 5-point Likert scale for each of the questions on the activity effectiveness questionnaire *Note:* Mean responses and standard deviation on the 5-point Likert scale for each of the questions on the activity effectiveness questionnaire.

Results from the activity questionnaire were analyzed using non-parametric Wilcoxon Signed Rank tests for each of the questions (see Figure 1). The comparison value for these analyses was 3 (neither agree nor disagree). Z-scores for these questions ranged from 3.61 to 3.91 and all *p* values were < .001.

## Discussion

GSR is a widely used technique for assessing sympathetic autonomic nervous system activity. Here we report the results of an easy and inexpensive lab activity for assessing responses to negative emotionally arousing stimuli. This simple activity requires relatively little time to complete and is relevant to many neuroscientific subdisciplines, including Affective, Behavioral, and Forensic Neuroscience.

Results of the activity effectiveness questionnaire indicated that the activity was very well received. Students felt that participating was a valuable experience that should be used in future classes. They reported that it was beneficial to discuss the activity and how the data were analyzed and that they gained a better understanding of how GSR reflects sympathetic nervous system arousal and how this technique is used.

The data reported here were obtained by testing one participant at a time using negative emotionally arousing stimuli. However, this activity could easily be modified in several ways. For example, with the acquisition of multiple GSR units, students could be divided into small groups, provided with sets of stimuli, and allowed to collect data from each other to provide a more direct and hands-on approach to the activity. While the activity reported here used only negative images, and did not differentiate between negative faces and other negative pictures, activities could be developed that would allow students to examine only one type of stimulus (e.g., threatening animals), to compare different types of stimuli (e.g., emotional faces of different racial/ethnic groups), to examine GSR for stimuli with a positive valence, to assess GSR over longer periods of stimulus exposure, etc. Faculty interested in developing a series of labs related to stress and emotional arousal might consider the addition of activities involving hypermnesia and reminiscence for emotionally arousing words or spatial memory for emotionally arousing pictures (Flint, 2004). Examination of blood glucose or cortisol levels, as indicators of stress, emotional arousal, or other variables related to classroom performance, might also be incorporated to expand relevant hands-on experiences for students (Dimolareva et al., 2018; Flint, 2004; Kalman & Grahn, 2004; Park et al., 2023; Preuβ et al., 2010; Silva et al., 2020; Snopkowski et al., 2019). The flexibility of this activity is a particular strength, as it allows interested faculty to modify the stimuli and/or procedures to meet the needs of their course and to connect the activity with their course material in a meaningful way.
